# Supplementary material for: The brain selectively allocates energy to functional brain networks under cognitive control
Source: Sci Rep. 2024 Dec 30;14:32032. doi: 10.1038/s41598-024-83696-7 (PMC11686059; doi:10.1038/s41598-024-83696-7)
Supplement: Supplementary file 1 — Supplementary Material 1 [file 41598_2024_83696_MOESM1_ESM.pdf]

Supplementary Material

The brain selectively allocates energy to functional brain networks under cognitive control

Majid Saberi[1, 2*], Jenny R. Rieck[3], Shamim Golafshan[4], Cheryl L. Grady[3, 5, 6], Bratislav Misic[7, 8], Benjamin T. Dunkley[1, 9, 10], Ali Khatibi[11, 12 *]

1- Neurosciences & Mental Health Program, The Hospital for Sick Children Research Institute, Toronto, Canada
2- Headache and Orofacial Pain Effort (H.O.P.E.) Laboratory, Department of Biologic and Materials Sciences & Prosthodontics, University of Michigan School of Dentistry, Ann Arbor, Michigan, USA
3- Rotman Research Institute, Baycrest Health Sciences, Toronto, M6A 2E1, Canada
4- Department of Psychology, Florida International University, Miami, FL, USA
5- Psychology, University of Toronto, 100 St. George Street, Toronto, ON, M5S 3G3, Canada
6- Psychiatry, University of Toronto, Toronto, M5T 1R8, Canada
7- Department of Neurology and Neurosurgery, McGill University, Montreal, QC, Canada
8- McConnell Brain Imaging Centre, Montreal Neurological Institute and Hospital, Montreal, QC, Canada
9- Department of Diagnostic Imaging, The Hospital for Sick Children, Toronto, Canada
10- Department of Medical Imaging, University of Toronto, Toronto, Canada
11- Centre of Precision Rehabilitation for Spinal Pain (CPR Spine), School of Sport, Exercise and Rehabilitation Sciences, University of Birmingham, Birmingham, UK
12- Centre for Human Brain Health, University of Birmingham, Birmingham, UK

* **Corresponding authors**
Majid Saberi: majidsa@umich.edu
Ali Khatibi: m.khatibitabatabaei@bham.ac.uk

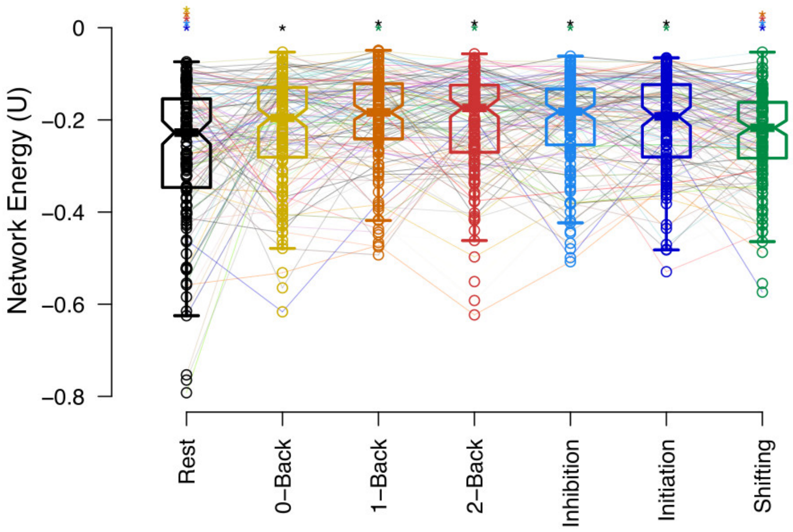

Figure S1. Energy of whole-brain network during cognitive control tasks and resting-state extracted based on Schaefer's atlas. Horizontal lines represent group-level medians, while notches on the boxes signify the 95% confidence interval for the median. Individual subjects are linked by lines, with task conditions differentiated by color. Colored asterisks above their respective conditions mark significant corrected p-values for non-parametric pair-wise comparisons between cognitive task conditions.

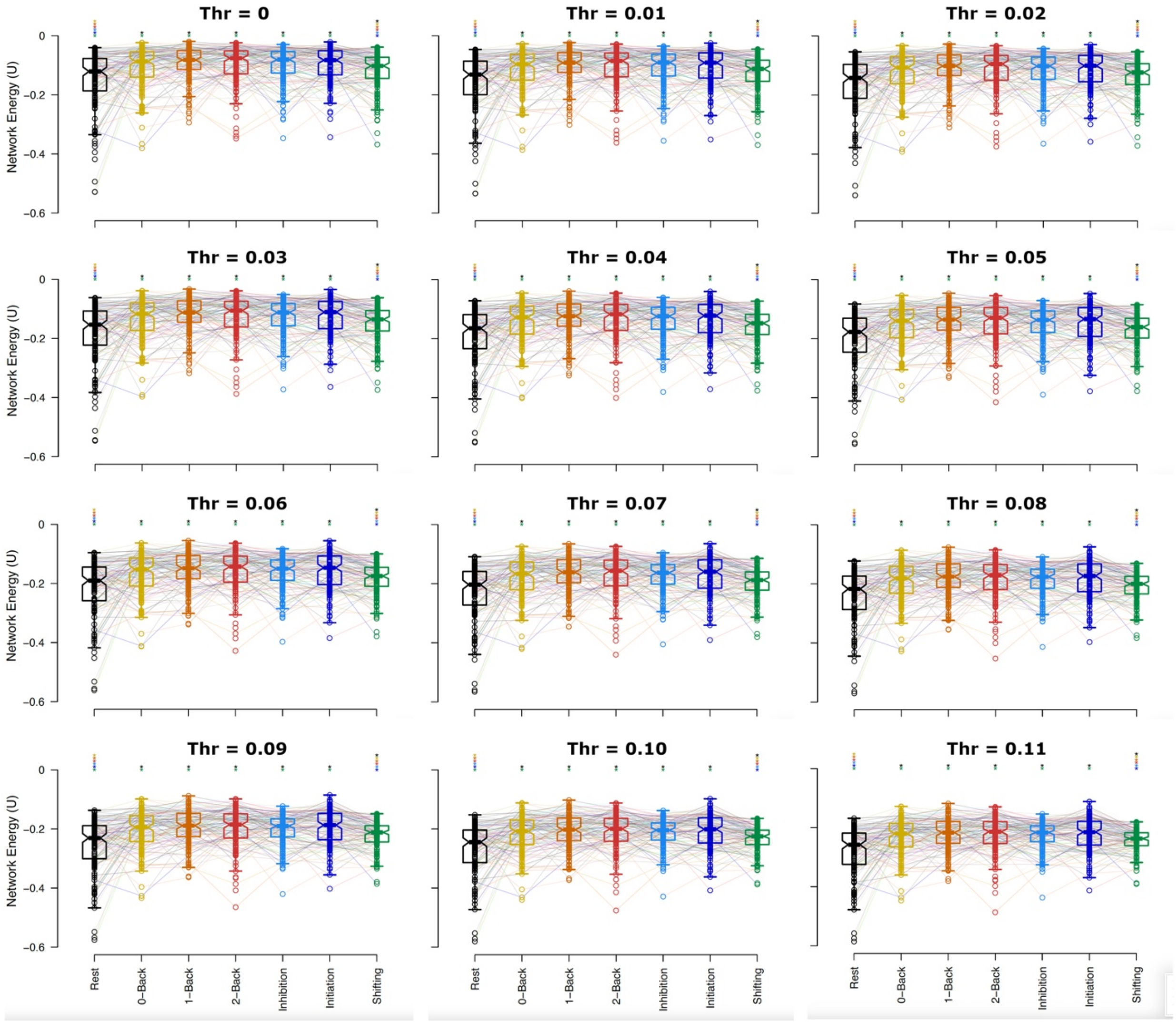

Figure S2. Effect of thresholding on the energy level of the whole-brain network during cognitive control tasks and resting-state, extracted using Power's atlas. Thresholds were applied to positive and negative connections with absolute correlation values less than the indicated thresholds before computing network energy. Horizontal lines represent group-level medians, and notches on the boxes indicate the 95% confidence intervals for the medians. Individual subjects are connected by lines, with task conditions distinguished by color. Colored asterisks above the respective conditions denote significant corrected p-values for non-parametric pairwise comparisons between cognitive task conditions.

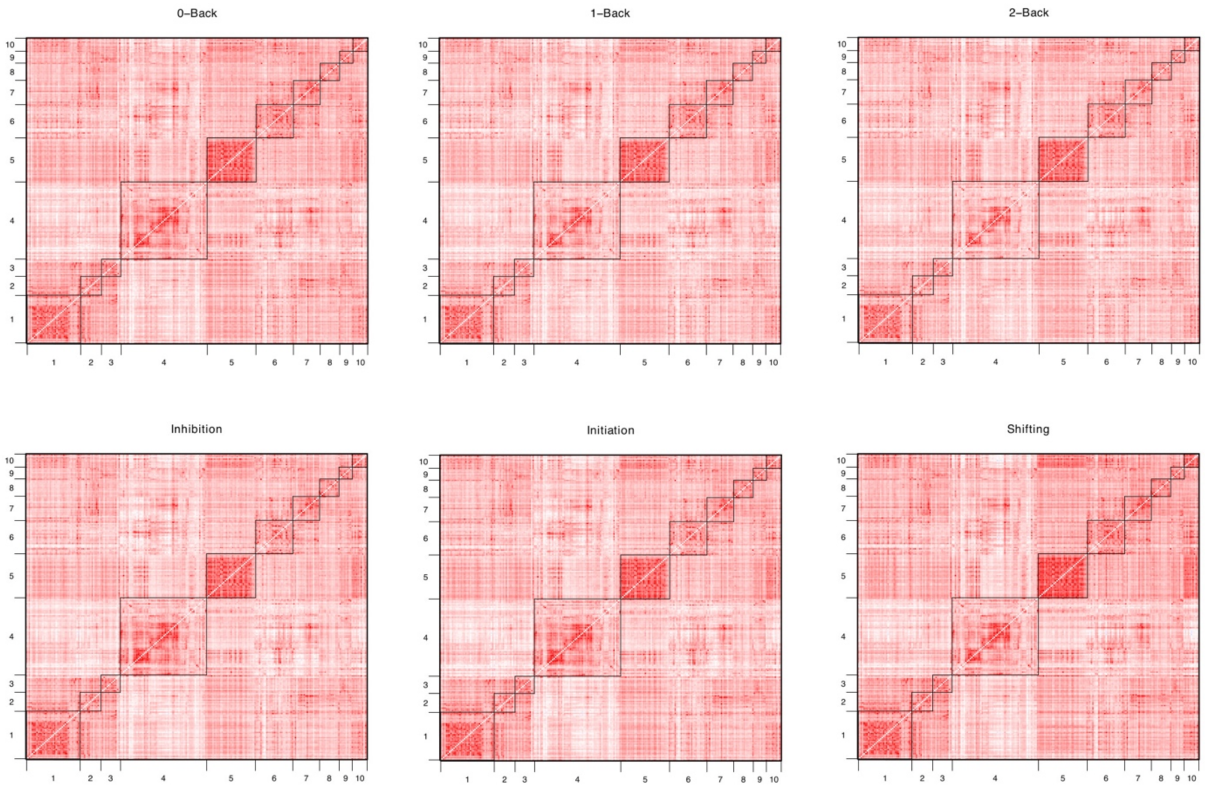

Figure S3. Group-average functional connectivity across all subjects for each task condition. The 229 Power's regions of interest were categorized into 10 canonical functional networks. Internetwork connections are represented by squares, with the intensity of colors indicating the strength of the group-average connections. Network labels are as follows: 1: Somatosensory, 2: Cingulate-Opercular, 3: Auditory, 4: Default Mode, 5: Visual, 6: Prefrontal, 7: Salience, 8: Subcortical, 9: Ventral Attention, 10: Dorsal Attention.

	Rest	0-Back	1-Back	2-Back	Inhibition	Initiation	Shifting
**Rest**	-	-	-	-	-	-	-
**0-Back**	0.018<br>(5.7E-5)	-	-	-	-	-	-
**1-Back**	0.033<br>(2.93E-7)	0.011<br>(0.370)	-	-	-	-	
**2-Back**	0.348<br>(3.48E-7)	0.004<br>(0.370)	0.001<br>(0.987)	-	-	-	-
**Inhibition**	0.031<br>(4.54E-7)	0.005<br>(0.525)	0.001<br>(0.820)	-0.001<br>(0.820)	-	-	-
**Initiation**	0.035<br>(6.22E-7)	0.001<br>(0.525)	-0.001<br>(0.808)	-0.005<br>(0.808)	-0.001<br>(0.987)	-	-
**Shifting**	0.013<br>(0.040)	-0.018<br>(0.031)	-0.020<br>(5.51E-4)	-0.015<br>(8.82 E-4)	-0.013<br>(0.001)	-0.025<br>(0.001)	-

Table S1. Pairwise Comparisons of Whole-Brain Network Energy Levels Across Various Cognitive Task Conditions. Cells indicate the median of paired differences among subjects, with p-values (in parentheses) from the Wilcoxon signed-rank test after applying multiple comparison correction. Highlighted cells represent comparisons with a corrected p-value less than 0.05.

	AUD	COP	DAN	DMN	FPC	SAL	SM	SC	VAN	VIS
**AUD**	-	-	-	-	-	-	-	-	-	-
**COP**	-0.046<br>(0.002)	-	-	-	-	-	-	-	-	-
**DAN**	-0.037<br>(0.326)	0.027<br>(0.067)	-	-	-	-	-	-	-	-
**DMN**	0.079<br>(4.19E-8)	0.129<br>(8.81E-18)	0.119<br>(1.45E-10)	-	-	-	-	-	-	-
**FPC**	0.072<br>(3.95E-6)	0.129<br>(2.27E-14)	0.097<br>(2.96E-8)	-0.010<br>(0.444)	-	-	-	-	-	-
**SAL**	0.030<br>(0.047)	0.079<br>(7.32E-8)	0.051<br>(0.002)	-0.051<br>(6.38E-5)	-0.040<br>(0.002)	-	-	-	-	-
**SM**	-0.052<br>(0.583)	-0.015<br>(0.001)	-0.029<br>(0.461)	-0.144<br>(1.38E-10)	-0.128<br>(2.96E-8)	-0.085<br>(0.008)	-	-	-	-
**SC**	-0.002<br>(0.014)	0.042<br>(0.958)	0.015<br>(0.141)	-0.084<br>(4.05E-12)	-0.081<br>(4.73E-10)	-0.040<br>(3.82E-5)	0.037<br>(0.014)	-	-	-
**VAN**	0.006<br>(0.434)	0.067<br>(2.28E-4)	0.054<br>(0.057)	-0.083<br>(3.82E-5)	-0.065<br>(9.39E-4)	-0.014<br>(0.423)	0.073<br>(0.233)	0.014<br>(0.002)	-	-
**VIS**	-0.108<br>(6.65E-10)	-0.057<br>(8.18E-4)	-0.080<br>(1.12E-6)	-0.197<br>(1.46E-28)	-0.183<br>(2.81E-24)	-0.148<br>(4.21E-17)	-0.047<br>(3.79E-11)	-0.108<br>(0.009)	-0.136<br>(1.19E-10)	-

Table S2. Pairwise Comparisons of Canonical Networks' Energies During Resting State. Cells indicate the median of paired differences among subjects, with p-values (in parentheses) from the Wilcoxon signed-rank test after applying multiple comparison correction. Highlighted cells represent comparisons with a corrected p-value less than 0.05.

	AUD	COP	DAN	DMN	FPC	SAL	SM	SC	VAN	VIS
**AUD**	-	-	-	-	-	-	-	-	-	-
**COP**	-0.015<br>(0.679)	-	-	-	-	-	-	-	-	-
**DAN**	-0.018<br>(0.252)	0.002<br>(0.679)	-	-	-	-	-	-	-	-
**DMN**	0.031<br>(8.68E-4)	0.05<br>(3.96E-5)	0.057<br>(2.41E-7)	-	-	-	-	-	-	-
**FPC**	-0.014<br>(0.776)	0.01<br>(0.846)	0.013<br>(0.465)	-0.045<br>(3.96E-5)	-	-		-	-	-
**SAL**	-0.003<br>(0.776)	0.004<br>(0.846)	0.012<br>(0.514)	-0.035<br>(8.71E-5)	-0.012<br>(0.944)	-	-	-	-	-
**SM**	-0.015<br>(0.498)	-0.005<br>(0.846)	-0.01<br>(0.846)	-0.059<br>(1.88E-5)	-0.012<br>(0.679)	-0.016<br>(0.679)	-	-	-	-
**SC**	-0.013<br>(0.114)	0.005<br>(0.558)	-0.003<br>(0.944)	-0.049<br>(2.12E-8)	-0.028<br>(0.373)	-0.02<br>(0.418)	0.000615<br>(0.775)	-	-	-
**VAN**	-0.008<br>(0.776)	0.01<br>(0.846)	0.016<br>(0.511)	-0.047<br>(2.34E-4)	-0.005<br>(0.944)	0.004<br>(0.944)	0.010308<br>(0.688)	0.008<br>(0.498)	-	-
**VIS**	-0.09<br>(3.44E-10)	-0.079<br>(9.13E-8)	-0.078<br>(2.31E-7)	-0.132<br>(1.31E-22)	-0.08<br>(9.33E-10)	-0.07585<br>(3.69E-9)	-0.067<br>(4.77E-7)	-0.074<br>(2.31E-7)	-0.076<br>(4.50E-8)	-

Table S3. Pairwise Comparisons of Canonical Networks' Energies During 0-Back Task. Cells indicate the median of paired differences among subjects, with p-values (in parentheses) from the Wilcoxon signed-rank test after applying multiple comparison correction. Highlighted cells represent comparisons with a corrected p-value less than 0.05.

	AUD	COP	DAN	DMN	FPC	SAL	SM	SC	VAN	VIS
**AUD**	-	-	-	-	-	-	-	-	-	-
**COP**	0.001 (0.858)	-	-	-	-	-	-	-	-	-
**DAN**	-0.019 (0.105)	-0.016 (0.154)	-	-	-	-	-	-	-	-
**DMN**	0.029 (0.001)	0.034 (0.001)	0.057 (1.17E-07)	-	-	-	-	-	-	-
**FPC**	-0.01 (0.150)	-0.012 (0.217)	0.004 (0.827)	-0.043 (4.73E-8)	-	-	-	-	-	-
**SAL**	-0.019 (0.150)	-0.013 (0.219)	-0.003 (0.827)	-0.052 (1.34E-7)	-0.006 (0.998)	-	-	-	-	-
**SM**	-0.028 (0.290)	-0.023 (0.319)	-0.002 (0.646)	-0.059 (1.93E-06)	-0.024 (0.858)	-0.007 (0.858)	-	-	-	-
**SC**	-0.013 (0.009)	-0.015 (0.017)	0.003 (0.563)	-0.041 (1.14E-10)	-0.005 (0.361)	0.001 (0.395)	0.015 (0.315)	-	-	-
**VAN**	-0.006 (0.467)	-0.011 (0.563)	0.008 (0.467)	-0.051 (3.89E-05)	0.001 (0.563)	0.002 (0.570)	0.016 (0.784)	-0.002 (0.150)	-	-
**VIS**	-0.086 (2.69E-11)	-0.077 (1.09E-10)	-0.06 (4.11E-07)	-0.131 (2.69E-24)	-0.08 (5.97E-09)	-0.07 (2.64E-8)	-0.059 (2.64E-8)	-0.08 (3.27E-6)	-0.074 (2.64E-8)	-

Table S4. Pairwise Comparisons of Canonical Networks' Energies During 1-Back Task. Cells indicate the median of paired differences among subjects, with p-values (in parentheses) from the Wilcoxon signed-rank test after applying multiple comparison correction. Highlighted cells represent comparisons with a corrected p-value less than 0.05.

	AUD	COP	DAN	DMN	FPC	SAL	SM	SC	VAN	VIS
**AUD**	-	-	-	-	-	-	-	-	-	-
**COP**	0.005 (0.405)	-	-	-	-	-	-	-	-	-
**DAN**	-0.013 (0.084)	-0.029 (0.011)	-	-	-	-	-	-	-	-
**DMN**	0.044 (1.22E-07)	0.038 (5.21E-05)	0.068 (1.05E-12)	-	-	-	-	-	-	-
**FPC**	-0.014 (0.340)	-0.019 (0.084)	0.006 (0.405)	-0.055 (4.69E-11)	-	-	-	-	-	-
**SAL**	-0.004 (0.938)	-0.003 (0.391)	0.012 (0.087)	-0.048 (2.51E-08)	0.006 (0.391)	-	-	-	-	-
**SM**	-0.010 (0.279)	-0.018 (0.057)	-0.001 (0.710)	-0.064 (2.01E-10)	-0.007 (0.785)	-0.017 (0.292)	-	-	-	-
**SC**	-0.017 (0.094)	-0.023 (0.013)	0.001 (0.949)	-0.073 (1.93E-12)	-0.018 (0.441)	-0.023 (0.104)	0.004 (0.726)	-	-	-
**VAN**	-0.002 (0.938)	-0.022 (0.405)	0.013 (0.173)	-0.053 (8.20E-07)	0.007 (0.456)	-0.008 (0.984)	0.015 (0.391)	0.015 (0.217)	-	-
**VIS**	-0.088 (8.77E-13)	-0.095 (7.13E-14)	-0.071 (4.71E-09)	-0.140 (1.24E-28)	-0.075 (1.31E-11)	-0.089 (3.50E-13)	-0.064 (5.92E-09)	-0.062 (4.5E-09)	-0.087 (4.00E-10)	-

Table S5. Pairwise Comparisons of Canonical Networks' Energies During 2-Back Task. Cells indicate the median of paired differences among subjects, with p-values (in parentheses) from the Wilcoxon signed-rank test after applying multiple comparison correction. Highlighted cells represent comparisons with a corrected p-value less than 0.05.

	AUD	COP	DAN	DMN	FPC	SAL	SM	SC	VAN	VIS
**AUD**	-	-	-	-	-	-	-	-	-	-
**COP**	-0.012 (0.533)	-	-	-	-	-	-	-	-	-
**DAN**	-0.021 (0.111)	-0.006 (0.461)	-	-	-	-	-	-	-	-
**DMN**	0.031 (8.84E-4)	0.047 (1.36E-05)	0.057 (4.97E-08)	-	-	-	-	-	-	-
**FPC**	0.029 (0.046)	0.037 (0.002)	0.044 (2.93E-05)	-0.013 (0.229)	-	-	-	-	-	-
**SAL**	-0.004 (0.515)	0.002 (0.992)	0.010 (0.366)	-0.056 (5.08E-06)	-0.029 (0.001)	-	-	-	-	-
**SM**	-0.031 (0.218)	-0.015 (0.583)	-0.001 (0.749)	-0.062 (3.29E-07)	-0.054 (1.18E-4)	-0.015 (0.533)	-	-	-	-
**SC**	-0.025 (0.020)	-0.006 (0.156)	-0.011 (0.704)	-0.064 (3.39E-10)	-0.048 (8.61E-07)	-0.018 (0.156)	0.001 (0.488)	-	-	-
**VAN**	-0.009 (0.747)	0.007 (0.813)	0.002 (0.322)	-0.046 (9.32E-05)	-0.029 (0.008)	0.0003 (0.749)	0.024 (0.420)	0.012 (0.097)	-	-
**VIS**	-0.088 (2.60E-09)	-0.070 (6.83E-08)	-0.059 (1.16E-06)	-0.125 (4.87E-23)	-0.114 (1.96E-18)	-0.068 (1.03E-08)	-0.053 (4.09E-07)	-0.057 (4.96E-06)	-0.073 (4.32E-08)	-

Table S6. Pairwise Comparisons of Canonical Networks' Energies During Inhibition Condition of Go/No-go Task. Cells indicate the median of paired differences among subjects, with p-values (in parentheses) from the Wilcoxon signed-rank test after applying multiple comparison correction. Highlighted cells represent comparisons with a corrected p-value less than 0.05.

	AUD	COP	DAN	DMN	FPC	SAL	SM	SC	VAN	VIS
**AUD**	-	-	-	-	-	-	-	-	-	-
**COP**	-0.007 (0.384)	-	-	-	-	-	-	-	-	-
**DAN**	-0.012 (0.367)	-0.003 0.929	-	-	-	-	-	-	-	-
**DMN**	0.033 (5.51E-05)	0.044 (8.02E-07)	0.058 (1.74E-07)	-	-	-	-	-	-	-
**FPC**	0.025 (0.024)	0.036 (8.29E-04)	0.037 (5.58E-04)	-0.014 (0.155)	-	-	-	-	-	-
**SAL**	-0.004 (0.926)	0.012 (0.517)	0.005 (0.537)	-0.040 (4.43E-05)	-0.020 (0.016)	-	-	-	-	-
**SM**	-0.0366 (0.537)	-0.011 (0.826)	-0.013 (0.778)	-0.06896 (6.53E-06)	-0.06135 (0.003)	-0.036 (0.638)	-	-	-	-
**SC**	-0.025 (0.021)	0.009 (0.289)	0.007 (0.232)	-0.055 (2.90E-11)	-0.032 (8.02E-07)	-0.018 (0.044)	0.012 (0.122)	-	-	-
**VAN**	0.002 (0.617)	0.007 (0.848)	0.014 (0.860)	-0.047 (8.77E-06)	-0.026 (0.004)	-0.013 (0.623)	0.028 (0.929)	0.008 (0.204)	-	-
**VIS**	-0.085 (6.13E-10)	-0.077 (4.16E-07)	-0.076 (1.34E-07)	-0.129 (2.96E-23)	-0.121 (5.57E-17)	-0.081 (1.87E-09)	-0.042 (1.63E-07)	-0.073 (2.41E-05)	-0.091 (1.63E-07)	-

Table S7. Pairwise Comparisons of Canonical Networks' Energies During Initiation Condition of Go/No-go Task. Cells indicate the median of paired differences among subjects, with p-values (in parentheses) from the Wilcoxon signed-rank test after applying multiple comparison correction. Highlighted cells represent comparisons with a corrected p-value less than 0.05.

	AUD	COP	DAN	DMN	FPC	SAL	SM	SC	VAN	VIS
**AUD**	-	-	-	-	-	-	-	-	-	-
**COP**	-0.006 (0.261)	-	-	-	-	-	-	-	-	-
**DAN**	-0.079 (7.47E-08)	-0.058 (1.74E-05)	-	-	-	-	-	-	-	-
**DMN**	0.067 (1.82E-11)	0.094 (2.29E-15)	0.154 (1.76E-30)	-	-	-	-	-	-	-
**FPC**	0.005 (0.736)	0.015 (0.145)	0.077 (1.76E-10)	-0.071 (1.77E-12)	-	-	-	-	-	-
**SAL**	-0.005 (0.679)	0.018 (0.408)	0.059 (3.48E-08)	-0.085 (2.21E-14)	-0.013 (0.489)	-	-	-	-	-
**SM**	-0.027 (0.009)	0.001 (0.153)	0.056 (0.002)	-0.092 (3.13E-21)	-0.022 (0.0008)	-0.004 (0.010)	-	-	-	-
**SC**	-0.042 (0.170)	-0.009 (0.894)	0.031 (1.24E-06)	-0.105 (5.37E-19)	-0.033 (0.099)	-0.031 (0.325)	-0.021 (0.100)	-	-	-
**VAN**	0.005 (0.357)	0.034 (0.035)	0.075 (1.41E-10)	-0.066 (5.50E-09)	0.005 (0.446)	0.006 (0.157)	0.031 (1.87E-04)	0.047 (0.014)	-	-
**VIS**	-0.122 (5.37E-19)	-0.111 (1.34E-15)	-0.059 (2.77E-06)	-0.211 (4.91E-39)	-0.121 (8.14E-24)	-0.132 (6.14E-20)	-0.115 (1.87E-13)	-0.099 (8.23E-19)	-0.140 (2.24E-21)	-

Table S8. Pairwise Comparisons of Canonical Networks' Energies During Shifting Task. Cells indicate the median of paired differences among subjects, with p-values (in parentheses) from the Wilcoxon signed-rank test after applying multiple comparison correction. Highlighted cells represent comparisons with a corrected p-value less than 0.05.

	Rest	0-Back	1-Back	2-Back	Inhibition	Initiation	Shifting
**Rest**	-	-	-	-	-	-	-
**0-Back**	0.018 (5.7E-5)	-	-	-	-	-	-
**1-Back**	0.033 (2.93E-7)	0.011 (0.370)	-	-	-	-	
**2-Back**	0.348 (3.48E-7)	0.004 (0.370)	0.001 (0.987)	-	-	-	-
**Inhibition**	0.031 (4.54E-7)	0.005 (0.525)	0.001 (0.820)	-0.001 (0.820)	-	-	-
**Initiation**	0.035 (6.22E-7)	0.001 (0.525)	-0.001 (0.808)	-0.005 (0.808)	-0.001 (0.987)	-	-
**Shifting**	0.013 (0.040)	-0.018 (0.031)	-0.020 (5.51E-4)	-0.015 (8.82 E-4)	-0.013 (0.001)	-0.025 (0.001)	-

Table S9. Pairwise Comparisons of Auditory Network's Energy Across Cognitive Task Conditions. Cells indicate the median of paired differences among subjects, with p-values (in parentheses) from the Wilcoxon signed-rank test after applying multiple comparison correction. Highlighted cells represent comparisons with a corrected p-value less than 0.05.

	Rest	0-Back	1-Back	2-Back	Inhibition	Initiation	Shifting
**Rest**	-	-	-	-	-	-	-
**0-Back**	0.150 (3.21E-19)	-	-	-	-	-	-
**1-Back**	0.185 (4.26E-26)	0.037 (0.006)	-	-	-	-	
**2-Back**	0.190 (4.18E-28)	0.044 (0.001)	0.010 (0.631)	-	-	-	-
**Inhibition**	0.159 (3.38E-20)	-0.001 (0.874)	-0.024 (0.002)	-0.040 (3.80E-04)	-	-	-
**Initiation**	0.167 (3.38E-20)	0.006 (0.874)	-0.018 (0.005)	-0.044 (6.48E-04)	-0.001 (0.874)	-	-
**Shifting**	0.116 (4.15E-13)	-0.035 (0.002)	-0.061 (1.34E-08)	-0.080 (1.54E-10)	-0.033 (0.002)	-0.037 (0.002)	-

Table S10. Pairwise Comparisons of Cingulo-Opercular Network's Energy Across Cognitive Task Conditions. Cells indicate the median of paired differences among subjects, with p-values (in parentheses) from the Wilcoxon signed-rank test after applying multiple comparison correction. Highlighted cells represent comparisons with a corrected p-value less than 0.05.

	Rest	0-Back	1-Back	2-Back	Inhibition	Initiation	Shifting
**Rest**	-	-	-	-	-	-	-
**0-Back**	0.117 (2.17E-12)	-	-	-	-	-	-
**1-Back**	0.128 (1.73E-15)	0.018 (0.117)	-	-	-	-	
**2-Back**	0.121 (4.85E-17)	0.016 (0.130)	-0.001 0.803)	-	-	-	-
**Inhibition**	0.114 (7.38E-12)	-0.008 (0.643)	-0.027 (0.036)	-0.029 (0.030)	-	-	-
**Initiation**	0.116 (8.59E-14)	0.008 (0.623)	-0.013 (0.287)	-0.014 (0.352)	0.009 (0.247)	-	-
**Shifting**	0.012 (0.153)	-0.088 (2.13E-11)	-0.099 (6.38E-15)	-0.112 (4.31E-17)	-0.080 (3.90E-11)	-0.107 (2.94E-13)	-

Table S11. Pairwise Comparisons of Dorsal Attention Network's Energy Across Cognitive Task Conditions. Cells indicate the median of paired differences among subjects, with p-values (in parentheses) from the Wilcoxon signed-rank test after applying multiple comparison correction. Highlighted cells represent comparisons with a corrected p-value less than 0.05.

	Rest	0-Back	1-Back	2-Back	Inhibition	Initiation	Shifting
**Rest**	-	-	-	-	-	-	-
**0-Back**	0.065 (8.37E-12)	-	-	-	-	-	-
**1-Back**	0.086 (6.60E-19)	0.019 (0.009)	-	-	-	-	
**2-Back**	0.096 (1.06E-21)	0.030 (1.38E-04)	0.008 (0.183)	-	-	-	-
**Inhibition**	0.065 (6.32E-11)	-0.005 (0.608)	-0.018 (0.001)	-0.029 (6.37E-06)	-	-	-
**Initiation**	0.076 (1.48E-13)	0.001 (0.587)	-0.010 (0.047)	-0.023 (8.45E-04)	0.011 (0.286)	-	-
**Shifting**	0.073 (3.15E-12)	0.002 (0.997)	-0.026 (0.006)	-0.027 (5.13E-05)	0.001 (0.621)	-0.007 (0.549)	-

Table S12. Pairwise Comparisons of Default Mode Network's Energy Across Cognitive Task Conditions. Cells indicate the median of paired differences among subjects, with p-values (in parentheses) from the Wilcoxon signed-rank test after applying multiple comparison correction. Highlighted cells represent comparisons with a corrected p-value less than 0.05.

	Rest	0-Back	1-Back	2-Back	Inhibition	Initiation	Shifting
**Rest**	-	-	-	-	-	-	-
**0-Back**	0.030<br>(0.004)	-	-	-	-	-	-
**1-Back**	0.040<br>(7.09E-05)	0.012<br>(0.298)	-	-	-	-	
**2-Back**	0.035<br>(1.38E-05)	0.012<br>(0.189)	0.001<br>(0.752)	-	-	-	-
**Inhibition**	0.056<br>(9.54E-08)	0.030<br>(0.019)	0.017<br>(0.189)	0.012<br>(0.288)	-	-	-
**Initiation**	0.050<br>(3.18E-08)	0.027<br>(0.003)	0.021<br>(0.059)	0.023<br>(0.080)	0.001<br>(0.453)	-	-
**Shifting**	0.008<br>(0.453)	-0.013<br>(0.024)	-0.034<br>(4.55E-04)	-0.030<br>(6.22E-05)	-0.049<br>(3.02E-07)	-0.064<br>(9.54E-08)	-

Table S13. Pairwise Comparisons of Frontoparietal Network's Energy Across Cognitive Task Conditions. Cells indicate the median of paired differences among subjects, with p-values (in parentheses) from the Wilcoxon signed-rank test after applying multiple comparison correction. Highlighted cells represent comparisons with a corrected p-value less than 0.05.

	Rest	0-Back	1-Back	2-Back	Inhibition	Initiation	Shifting
**Rest**	-	-	-	-	-	-	-
**0-Back**	0.076<br>(2.87E-08)	-	-	-	-	-	-
**1-Back**	0.087<br>(2.81E-11)	0.010<br>(0.338)	-	-	-	-	
**2-Back**	0.100<br>(1.74E-15)	0.022<br>(0.034)	0.007<br>(0.302)	-	-	-	-
**Inhibition**	0.065<br>(4.35E-08)	-0.010<br>(0.585)	-0.021<br>(0.103)	-0.034<br>(0.003)	-	-	-
**Initiation**	0.078<br>(1.11E-09)	0.002<br>(0.615)	-0.010<br>(0.585)	-0.018<br>(0.076)	0.015<br>(0.346)	-	-
**Shifting**	0.040<br>(0.001)	-0.034<br>(0.004)	-0.048<br>(7.93E-05)	-0.058<br>(4.35E-08)	-0.030<br>(0.017)	-0.036<br>(0.002)	-

Table S14. Pairwise Comparisons of Salience Network's Energy Across Cognitive Task Conditions. Cells indicate the median of paired differences among subjects, with p-values (in parentheses) from the Wilcoxon signed-rank test after applying multiple comparison correction. Highlighted cells represent comparisons with a corrected p-value less than 0.05.

	Rest	0-Back	1-Back	2-Back	Inhibition	Initiation	Shifting
**Rest**	-	-	-	-	-	-	-
**0-Back**	0.114<br>(4.56E-14)	-	-	-	-	-	-
**1-Back**	0.122<br>(2.07E-16)	0.013<br>(0.217)	-	-	-	-	
**2-Back**	0.158<br>(3.60E-18)	0.025<br>(0.043)	0.005<br>(0.573)	-	-	-	-
**Inhibition**	0.119<br>(1.88E-12)	-0.008<br>(0.415)	-0.027<br>(0.025)	-0.025<br>(0.004)	-	-	-
**Initiation**	0.100<br>(1.88E-12)	-0.009<br>(0.573)	-0.017<br>(0.053)	-0.022<br>(0.012)	-0.001<br>(0.771)	-	-
**Shifting**	0.107<br>(2.28E-09)	-0.036<br>(0.004)	-0.045<br>(2.75E-05)	-0.056<br>(4.91E-07)	-0.016<br>(0.033)	-0.024<br>(0.025)	-

Table S15. Pairwise Comparisons of Somatomotor Network's Energy Across Cognitive Task Conditions. Cells indicate the median of paired differences among subjects, with p-values (in parentheses) from the Wilcoxon signed-rank test after applying multiple comparison correction. Highlighted cells represent comparisons with a corrected p-value less than 0.05.

	Rest	0-Back	1-Back	2-Back	Inhibition	Initiation	Shifting
**Rest**	-	-	-	-	-	-	-
**0-Back**	0.103 (2.38E-11)	-	-	-	-	-	-
**1-Back**	0.121 (1.76E-17)	0.036 (0.088)	-	-	-	-	
**2-Back**	0.114 (4.50E-17)	0.026 (0.153)	-0.010 (0.775)	-	-	-	-
**Inhibition**	0.092 (1.82E-12)	-0.001 (0.666)	-0.036 (0.017)	-0.025 (0.028)	-	-	-
**Initiation**	0.102 (1.99E-12)	0.001 (0.676)	-0.024 (0.214)	-0.009 (0.333)	0.004 (0.353)	-	-
**Shifting**	0.053 (1.41E-04)	-0.059 (7.72E-05)	-0.085 (9.30E-10)	-0.075 (3.25E-09)	-0.044 (7.91E-05)	-0.057 (3.55E-06)	-

Table S16. Pairwise Comparisons of Subcortical Network's Energy Across Cognitive Task Conditions. Cells indicate the median of paired differences among subjects, with p-values (in parentheses) from the Wilcoxon signed-rank test after applying multiple comparison correction. Highlighted cells represent comparisons with a corrected p-value less than 0.05.

	Rest	0-Back	1-Back	2-Back	Inhibition	Initiation	Shifting
**Rest**	-	-	-	-	-	-	-
**0-Back**	0.079 (2.38E-11)	-	-	-	-	-	-
**1-Back**	0.107 (1.76E-17)	0.024 (0.088)	-	-	-	-	
**2-Back**	0.115 (4.50E-17)	0.022 (0.153)	0.007 (0.775)	-	-	-	-
**Inhibition**	0.089 (1.82E-12)	-0.011 (0.666)	-0.009 (0.017)	-0.024 (0.028)	-	-	-
**Initiation**	0.081 (1.99E-12)	0.010 (0.676)	-0.008 (0.214)	-0.026 (0.333)	0.010 (0.353)	-	-
**Shifting**	0.065 (1.41E-04)	-0.021 (7.72E-05)	-0.026 (9.30E-10)	-0.041 (3.25E-09)	-0.009 (7.91E-05)	-0.011 (3.55E-06)	-

Table S17. Pairwise Comparisons of Ventral Attention Network's Energy Across Cognitive Task Conditions. Cells indicate the median of paired differences among subjects, with p-values (in parentheses) from the Wilcoxon signed-rank test after applying multiple comparison correction. Highlighted cells represent comparisons with a corrected p-value less than 0.05.

	Rest	0-Back	1-Back	2-Back	Inhibition	Initiation	Shifting
**Rest**	-	-	-	-	-	-	-
**0-Back**	0.152 (9.20E-16)	-	-	-	-	-	-
**1-Back**	0.156 (5.35E-19)	0.016 (0.238)	-	-	-	-	
**2-Back**	0.150 (4.00E-20)	0.016 (0.182)	0.004 (0.940)	-	-	-	-
**Inhibition**	0.141 (1.28E-16)	-0.001 (0.940)	-0.012 (0.247)	-0.019 (0.238)	-	-	-
**Initiation**	0.135 (2.32E-15)	0.004 (0.926)	-0.005 (0.254)	-0.015 (0.247)	-0.002 (0.941)	-	-
**Shifting**	0.073 (1.39E-05)	-0.076 (5.21E-08)	-0.108 (1.51E-11)	-0.088 (4.81E-13)	-0.070 (9.29E-09)	-0.079 (4.96E-08)	-

Table S18. Pairwise Comparisons of Visual Network's Energy Across Cognitive Task Conditions. Cells indicate the median of paired differences among subjects, with p-values (in parentheses) from the Wilcoxon signed-rank test after applying multiple comparison correction. Highlighted cells represent comparisons with a corrected p-value less than 0.05.

	AUD	COP	DAN	DMN	FPC	SAL	SM	SC	VAN	VIS
**AUD**	-	-	-	-	-	-	-	-	-	-
**COP**	0.042 (0.011)	-	-	-	-	-	-	-	-	-
**DAN**	0.011 (0.601)	-0.023 (0.059)	-	-	-	-	-	-	-	-
**DMN**	-0.037 (0.062)	-0.084 (1.27E-07)	-0.053 (0.016)	-	-	-	-	-	-	-
**FPC**	-0.067 (1.53E-04)	-0.117 (3.35E-11)	-0.090 (3.37E-05)	-0.036 (0.007)	-	-	-	-	-	-
**SAL**	-0.027 (0.116)	-0.065 (5.84E-06)	-0.039 (0.030)	-0.004 (0.999)	0.040 (0.034)	-	-	-	-	-
**SM**	0.044 (0.780)	-0.006 (0.002)	0.028 (0.393)	0.065 (0.063)	0.097 (2.94E-04)	0.073 (0.166)	-	-	-	-
**SC**	-0.011 (0.169)	-0.048 (0.523)	-0.021 (0.304)	0.029 (0.003)	0.072 (7.83E-07)	0.017 (0.003)	-0.042 (0.079)	-	-	-
**VAN**	-0.012 (0.448)	-0.066 (0.001)	-0.037 (0.258)	0.029 (0.425)	0.052 (0.006)	0.031 (0.448)	-0.052 (0.609)	0.0048 (0.031)	-	-
**VIS**	0.034 (0.166)	-0.008 (0.559)	0.012 (0.321)	0.078 (4.02E-04)	0.124 (7.20E-07)	0.070 (0.001)	-0.011 (0.051)	0.045 (0.765)	0.039 (0.036)	-

Table S19. Pairwise Comparisons of Networks' Energy Alterations in Transition from Rest to 0-Back Task. Cells indicate the median of paired differences among subjects, with p-values (in parentheses) from the Wilcoxon signed-rank test after applying multiple comparison correction. Highlighted cells represent comparisons with a corrected p-value less than 0.05.

	AUD	COP	DAN	DMN	FPC	SAL	SM	SC	VAN	VIS
**AUD**	-	-	-	-	-	-	-	-	-	-
**COP**	0.156 (2.44E-23)	-	-	-	-	-	-	-	-	-
**DAN**	0.105 (7.39E-11)	-0.036 (0.004)	-	-	-	-	-	-	-	-
**DMN**	0.062 (1.41E-09)	-0.085 (4.11E-10)	-0.049 (0.024)	-	-	-	-	-	-	-
**FPC**	0.016 (0.155)	-0.123 (9.63E-15)	-0.079 (2.82E-06)	-0.043 (6.03E-04)	-	-	-	-	-	-
**SAL**	0.062 (2.82E-06)	-0.093 (2.65E-09)	-0.059 (0.012)	-0.008 (0.590)	0.036 (0.007)	-	-	-	-	-
**SM**	0.112 (1.45E-14)	-0.001 (1.19E-04)	0.037 (0.820)	0.055 (0.005)	0.110 (1.29E-07)	0.061 (0.005)	-	-	-	-
**SC**	0.099 (1.92E-11)	-0.064 (0.141)	-0.012 (0.346)	0.034 (0.007)	0.076 (1.29E-07)	0.041 (0.002)	-0.022 (0.267)	-	-	-
**VAN**	0.072 (5.33E-07)	-0.060 (2.00E-05)	-0.034 (0.234)	0.024 (0.488)	0.066 (0.001)	0.023 (0.306)	-0.049 (0.243)	-0.024 (0.032)	-	-
**VIS**	0.106 (1.85E-11)	-0.009 (0.103)	0.023 (0.381)	0.075 (0.003)	0.109 (2.87E-07)	0.078 (0.001)	0.002 (0.234)	0.034 (0.727)	0.042 (0.039)	-

Table S20. Pairwise Comparisons of Networks' Energy Alterations in Transition from Rest to 1-Back Task. Cells indicate the median of paired differences among subjects, with p-values (in parentheses) from the Wilcoxon signed-rank test after applying multiple comparison correction. Highlighted cells represent comparisons with a corrected p-value less than 0.05.

	AUD	COP	DAN	DMN	FPC	SAL	SM	SC	VAN	VIS
**AUD**	-	-	-	-	-	-	-	-	-	-
**COP**	0.117 1.17E-09)	-	-	-	-	-	-	-	-	-
**DAN**	0.051 (0.006)	-0.045 (0.004)	-	-	-	-	-	-	-	-
**DMN**	0.016 (0.088)	-0.085 (1.29E-08)	-0.047 (0.124)	-	-	-	-	-	-	-
**FPC**	-0.031 (0.044)	-0.145 (2.94E-15)	-0.085 (5.54E-06)	-0.044 (3.55E-05)	-	-	-	-	-	-
**SAL**	0.013 (0.116)	-0.083 (2.03E-08)	-0.039 (0.115)	-0.001 (0.884)	0.052 (1.66E-04)	-	-	-	-	-
**SM**	0.084 (0.016)	-0.027 (2.66E-05)	0.036 (0.508)	0.050 (0.203)	0.104 (8.17E-06)	0.073 (0.267)	-	-	-	-
**SC**	0.036 (9.03E-05)	-0.068 (0.203)	-0.009 (0.172)	0.015 (0.004)	0.067 (1.68E-08)	0.032 (0.004)	-0.020 (0.053)	-	-	-
**VAN**	0.030 (0.142)	-0.068 (1.02E-04)	-0.030 (0.294)	0.011 (0.656)	0.067 (0.001)	0.002 (0.655)	-0.043 (0.655)	-0.014 (0.023)	-	-
**VIS**	0.080 (8.25E-05)	-0.024 (0.041)	0.021 (0.339)	0.057 (0.002)	0.117 (1.16E-08)	0.059 (0.003)	-0.022 (0.073)	0.039 (0.655)	0.032 (0.053)	-

Table S21. Pairwise Comparisons of Networks' Energy Alterations in Transition from Rest to 2-Back Task. Cells indicate the median of paired differences among subjects, with p-values (in parentheses) from the Wilcoxon signed-rank test after applying multiple comparison correction. Highlighted cells represent comparisons with a corrected p-value less than 0.05.

	AUD	COP	DAN	DMN	FPC	SAL	SM	SC	VAN	VIS
**AUD**	-	-	-	-	-	-	-	-	-	-
**COP**	0.036 (0.065)	-	-	-	-	-	-	-	-	-
**DAN**	0.017 (0.912)	-0.019 (0.159)	-	-	-	-	-	-	-	-
**DMN**	-0.039 (0.009)	-0.076 (3.83E-06)	-0.042 (0.021)	-	-	-	-	-	-	-
**FPC**	-0.041 (0.002)	-0.097 (3.83E-06)	-0.059 (0.003)	-0.006 (0.289)	-	-	-	-	-	-
**SAL**	-0.037 (0.037)	-0.067 (7.32E-05)	-0.039 (0.045)	0.003 (0.890)	0.016 (0.304)	-	-	-	-	-
**SM**	0.023 (0.306)	-0.004 (0.003)	0.013 (0.299)	0.034 (0.217)	0.056 (0.059)	0.046 (0.304)	-	-	-	-
**SC**	-0.013 (0.632)	-0.046 (0.507)	-0.021 (0.686)	0.026 (0.027)	0.039 (0.004)	0.017 (0.037)	-0.025 (0.162)	-	-	-
**VAN**	-0.022 (0.178)	-0.062 (0.003)	-0.023 (0.191)	0.009 (0.599)	0.015 (0.268)	0.012 (0.686)	-0.059 (0.706)	-0.004 (0.095)	-	-
**VIS**	0.028 (0.432)	-0.020 (0.412)	0.005 (0.545)	0.058 (0.001)	0.052 (2.08E-04)	0.043 (0.003)	0.003 (0.068)	0.051 (0.891)	0.032 (0.046)	-

Table S22. Pairwise Comparisons of Networks' Energy Alterations in Transition from Rest to Inhibition Condition of Go/No-go Task. Cells indicate the median of paired differences among subjects, with p-values (in parentheses) from the Wilcoxon signed-rank test after applying multiple comparison correction. Highlighted cells represent comparisons with a corrected p-value less than 0.05.

	AUD	COP	DAN	DMN	FPC	SAL	SM	SC	VAN	VIS
**AUD**	-	-	-	-	-	-	-	-	-	-
**COP**	0.037 (0.026)	-	-	-	-	-	-	-	-	-
**DAN**	-0.002 (0.647)	-0.036 (0.013)	-	-	-	-	-	-	-	-
**DMN**	-0.042 (0.002)	-0.086 (1.67E-09)	-0.038 (0.034)	-	-	-	-	-	-	-
**FPC**	-0.050 (0.002)	-0.086 (6.98E-09)	-0.047 (0.018)	0.002 (0.559)	-	-	-	-	-	-
**SAL**	-0.034 (0.009)	-0.076 (1.16E-07)	-0.034 (0.057)	0.001 (0.884)	0.010 (0.631)	-	-	-	-	-
**SM**	0.024 (0.224)	-0.005 (7.87E-05)	0.034 (0.612)	0.048 (0.022)	0.060 (0.010)	0.076 (0.081)	-	-	-	-
**SC**	-0.018 (0.559)	-0.050 (0.221)	-0.003 (0.315)	0.041 (0.004)	0.036 (0.002)	0.029 (0.006)	-0.01519 (0.171)	-	-	-
**VAN**	-0.014 (0.232)	-0.058 (8.42E-04)	-0.026 (0.512)	0.022 (0.141)	0.033 (0.081)	0.027 (0.221)	-0.050 (0.754)	-0.001 (0.097)	-	-
**VIS**	0.031 (0.171)	-0.011 (0.483)	0.033 (0.084)	0.056 (1.23E-06)	0.064 (1.23E-06)	0.067 (1.21E-05)	-0.001 (0.005)	0.049 (0.572)	0.058 (0.010)	-

Table S23. Pairwise Comparisons of Networks' Energy Alterations in Transition from Rest to Initiation Condition of Go/No-go Task. Cells indicate the median of paired differences among subjects, with p-values (in parentheses) from the Wilcoxon signed-rank test after applying multiple comparison correction. Highlighted cells represent comparisons with a corrected p-value less than 0.05.

	AUD	COP	DAN	DMN	FPC	SAL	SM	SC	VAN	VIS
**AUD**	-	-	-	-	-	-	-	-	-	-
**COP**	0.033 (0.018)	-	-	-	-	-	-	-	-	-
**DAN**	-0.045 (0.002)	-0.077 (1.68E-06)	-	-	-	-	-	-	-	-
**DMN**	-0.008 (0.800)	-0.036 (0.002)	0.044 (0.002)	-	-	-	-	-	-	-
**FPC**	-0.055 (4.49E-06)	-0.101 (1.64E-11)	-0.014 (0.434)	-0.05 (1.05E-06)	-	-	-	-	-	-
**SAL**	-0.050 (0.025)	-0.069 (2.36E-06)	0.017 (0.314)	-0.025 (0.025)	0.024 (0.017)	-	-	-	-	-
**SM**	0.050 (0.063)	0.001 (3.85E-06)	0.081 (0.114)	0.022 (0.062)	0.112 (0.001)	0.049 (0.650)	-	-	-	-
**SC**	-0.039 (0.118)	-0.067 (0.800)	0.025 (2.42E-05)	-0.022 (0.063)	0.039 (3.59E-08)	-0.005 (3.99E-04)	-0.047 (0.001)	-	-	-
**VAN**	-0.002 (0.800)	-0.031 (0.018)	0.033 (0.007)	-0.001 (0.982)	0.061 (1.93E-05)	0.031 (0.051)	-0.050 (0.098)	0.021 (0.096)	-	-
**VIS**	-0.017 (0.672)	-0.035 (0.007)	0.040 (0.029)	-0.006 (0.800)	0.057 (3.79E-04)	0.026 (0.098)	-0.048 (0.201)	0.025 (0.033)	-0.001 (0.773)	-

Table S24. Pairwise Comparisons of Networks' Energy Alterations in Transition from Rest to Shifting Task. Cells indicate the median of paired differences among subjects, with p-values (in parentheses) from the Wilcoxon signed-rank test after applying multiple comparison correction. Highlighted cells represent comparisons with a corrected p-value less than 0.05.

Input Features	Accuracy	Kappa	Per Class Accuracy				
			Rest	Working Memory	Inhibitory Control	Cognitive Flexibility	Balanced Accuracy
Network Energy	0.577	0.24	0.601	0.78	0.339	0.367	0.527
Global Clustering Coefficient	0.464	0.135	0.218	0.82	0.276	0.015	0.332
Global Efficiency	0.52	0.268	0.52	0.802	0.205	0.3	0.457
Global Modularity	0.481	0.166	0.386	0.865	0.17	0.043	0.366
All Global Network Measures	0.61	0.415	0.697	0.794	0.43	0.33	0.563

Table S25. Performance of Cognitive Task Classification for Each Set of Input Features

Input Features	R^2	MAE
Network Energy	0.447	11.294
Global Clustering Coefficient	0.439	12.795
Global Efficiency	0.277	13.249
Global Modularity	0.394	12.442
All Global Network Measures	0.543	10.36

Table S26. Performance of Predicting Chronical Age for Each Set of Input Features.

Task	P-value	Median of Differences
Resting	<< .001	-4.05E-2
0-Back	<< .001	-3.37E-2
1-Back	<< .001	-3.39E-2
2-Back	<< .001	-3.17E-2
Inhibition	<< .001	-2.51E-2
Initiation	<< .001	-3.52E-2
Shifting	<< .001	-2.59E-2

Table S27. Pairwise Comparison of Energy between Whole-brain Networks during Cognitive Task Conditions and Their Corresponding Random Topology Networks

Task	ICC	Lower Band Confidence Interval	Upper Band Confidence Interval	P-value
Resting	0.94250563	0.9200982	0.95862918	2.89E-51
0-Back	0.915003	0.8818769	0.93883931	1.07E-40
1-Back	0.90190533	0.86367464	0.92941471	5.56E-37
2-Back	0.90647835	0.87002993	0.93270529	3.29E-38
Inhibition	0.92396689	0.89433431	0.94528939	1.19E-43
Initiation	0.89641186	0.8560402	0.92546182	1.35E-35
Shifting	0.93578342	0.91075613	0.95379213	3.21E-48

Table S28. Intraclass Correlation Statistics between Whole-brain Energy of Power's Atlas-based Networks and Schaefer's Atlas-based Networks.
